# Inhibiting uptake of extracellular vesicles derived from senescent bone marrow mesenchymal stem cells by muscle satellite cells attenuates sarcopenia

**DOI:** 10.1016/j.jot.2022.06.002

**Published:** 2022-07-06

**Authors:** Hanhao Dai, Wu Zheng, Jun Luo, Guoyu Yu, Chao Song, Yijing Wu, Jie Xu

**Affiliations:** a Shengli Clinical Medical College of Fujian Medical University, Fuzhou, 350000, People's Republic of China; b Department of Orthopedics, Fujian Provincial Hospital, Fujian Medical University, Fuzhou, 350000, People's Republic of China

**Keywords:** Sarcopenia, Osteoporosis, Senescence, Extracellular vesicles, CD81

## Abstract

**Objective:**

Osteoporosis is associated with senescence of bone marrow mesenchymal stem cells (BMSCs). Extracellular vesicles derived from senescent BMSCs (BMSC-EVs) could be uptaken by muscle satellite cells (SCs). We hypothesized that inhibiting the uptake of harmful BMSC-EVs by SCs could prevent patients with osteoporosis complicated with sarcopenia.

**Methods:**

Bioinformatics analysis was used to analyze senescent SCs. Myogenic potential of SCs was measured using myogenesis assay and immunofluorescence while muscle atrophy was measured using histological evaluation. And the interaction of cluster of differentiation (CD) 81 and the membrane proteins of SCs was verified using biotin pulldown assay.. CD81-specific siRNA (si-CD81) was used to knockdown CD81 and anti-CD81 antibody (anti-CD81 Ab) was used to block CD81.

**Results:**

Differentially expressed genes in senescent SCs were enriched in muscle cell differentiation. The myogenic potential of senescent SCs was significantly decreased. Senescent BMSC-EVs impaired myogenesis of SCs. CD81 on the surface of BMSC-EVs could bind to membrane proteins of SCs. Both knockdown of CD81 and blocking CD81 prevented the uptake of senescent BMSC-EVs by SCs, thus relieving harmful effects of senescent BMSC-EVs on muscle atrophy.

**Conclusion:**

Blocking CD81 on the surface of senescent BMSC-EVs attenuates sarcopenia in aged mice, which could be useful for prevention of sarcopenia in patients with osteoporosis in clinical practice.

**Translational potential of this article:**

Inhibiting uptake of extracellular vesicles derived from senescent bone marrow mesenchymal stem cells by muscle satellite cells can prevent muscle atrophy in aged mice and has potential for application in treating sarcopenia.

## 1 Introduction

Osteoporosis and sarcopenia are highly prevalent age-related musculoskeletal diseases in the elderly [1]. It is reported that decreased grip strength and lower limb strength are independent risk factors leading to falls and fractures in patients with osteoporosis [2]. And muscle atrophy is more effective than bone mineral density (BMD) and other risk factors in predicting fractures [3].

Among patients with osteoporosis, the incidence of sarcopenia is significantly increased [4,5]. Senescence and decreased osteogenic ability of bone marrow mesenchymal stem cells (BMSCs) are important factors in the development of osteoporosis [6,7]. Che et al. reported that the conditional medium (CM) derived from passage 15 BMSCs inhibited myogenic differentiation of C2C12 ​cells [8]. Fulzele et al. found that circulating extracellular vesicles (EVs) derived from oxidative-stress-induced senescent C2C12 ​cells induced senescence of BMSCs [9]. On the contrary, EVs produced under a variety of pathological conditions would be taken up by C2C12 ​cells, resulting in decreased proliferation and differentiation of C2C12 ​cells [10,11]. Thus, the susceptibility of osteoporosis patients to sarcopenia may be due to harmful EVs produced by senescent BMSCs that impair the muscle repair potential of muscle satellite cells (SCs).

EVs perform their functions by transporting cargo of non-coding RNAs, lipids and proteins between cell populations [12,13]. However, it is difficult to systematically intervene with the expression levels of these cargo *in vivo*. So, inhibiting the intake of senescent BMSC-derived EVs by SCs may help to solve this problem *in vivo*. The identified mechanisms of EV uptake include phagocytosis, receptor-mediated endocytosis, paracrine, and receptor-mediated membrane fusion [14]. Tetraspanin–integrin complexes on EVs were found to be an important component that regulated EV–cell interaction [15]. And Oguri reported that CD81, a tetraspanin abundant on the membranes of EVs, could form complexes with integrins [16]. Therefore, we hypothesized that inhibiting the uptake of harmful BMSC-EVs by SCs by blocking CD81 on the membrane of EVs could prevent and treat patients with osteoporosis complicated with sarcopenia.

In the present study, the effects of senescent BMSC-EVs on SCs were tested *in vitro* and *in vivo*. And gene knockdown and specific antibody blocking were used to block CD81 in the membranes of EVs to verify our hypothesis.

## 2 Materials and methods

### 2.1 Bioinformatics analysis

The Gene Expression Omnibus Series (GSE) GSE47401 microarray data [17] and the GSE116585 microarray data [18] were obtained from the Gene Expression Omnibus (GEO). Differentially expressed genes (DEGs) were identified by using the R package “limma” [19]. The cutoff criteria were |log2 fold change (FC)|> 0.5 and adjusted P ​< ​0.05. The heatmaps were formed by using R package “pheatmap” [20]. Functional enrichment analysis of Gene Ontology (GO) and Gene Set Enrichment Analysis (GSEA) for DEGs was performed by using R package “clusterprofiler” [21]. The cutoff criteria were P ​< ​0.05.

### 2.2 Animals

Thirty-five 4-month-old male C57/BL6 mice and twenty-five 20-month-old male C57/BL6 mice were provided by the Animal Experiment Center of Fujian Medical University. All animal experiments were approved by the Animal Ethics Committee of Fujian Medical University (NO. 2019-0134).

### 2.3 Isolation of SCs and BMSCs

To isolate SCs and BMSCs, the mice were sacrificed and disinfected by using 75% ethyl alcohol.

For SC isolation, hindlimb skeletal muscles, including musculus gastrocnemius (MG), tibialis anterior muscle (TA), and quadriceps femoris, were dissected. The muscle tissue was minced a smooth pulp and digested with 0.2% collagenase type II (C6885, Sigma, USA) for 2 ​h at 37 ​°C. Next, the homogenate was filtered using 70 ​μm nylon mesh cell strainer (431751, Corning, USA). Centrifuge the homogenate at 1500 ​rpm for 10 ​min and resuspend the pellet gently in 10 ​mL of growth medium. The solution was added into a dish and incubated at 37 ​°C in 5% CO_2_ for 2 ​h. Then the supernatant contenting SCs was added into a new dish to separate suspending SCs and adherent fibroblasts [22].

As for BMSC isolation, BMSCs were isolated by flushing the bone marrow from long bones of hind limbs [23]. Then BMSCs were cultured in high glucose Dulbecco's Modified Eagle Medium (DMEM) supplemented with 20% FBS and 1% penicillin/streptomycin.

### 2.4 Senescence associated β-galactosidase (β-gal) staining

Senescence associated β-gal staining was performed using a senescence β-gal staining kit (C0602, Beyotime, China) according to the manufacturer's instructions. Briefly, the adherent cells were fixed in 4% paraformaldehyde and stained with β-gal staining solution at room temperature (RT) overnight.

### 2.5 Myogenic differentiation assay

The SCs were seeded in 12-well plates. After 80–90% confluence, the growth medium was replaced by differentiation medium (high glucose DMEM medium supplemented with 2% horse serum and 1% penicillin/streptomycin). Myogenic differentiation assay was performed for 12 days.

### 2.6 Flow cytometric analysis

Flow cytometric analysis was performed using a mesenchymal stem cell (mouse) surface marker assay kit (MUXMX-09011, cyagen, China) on a FACS verse (Becton,Dickinson and Company (BD) fluorescence-activated cell sorting (FACS) Calibur, BD Biosciences, USA) according to the manufacturer's instructions.

### 2.7 Multilineage differentiation assay

Multilineage differentiation assay was performed using the OriCell^TM^ Mesenchymal Stem Cell Chondrogenic Differentiation Medium (GUXMX-90041, cyagen, China), OriCell^TM^ Bone Marrow Mesenchymal Stem Cell Osteogenic Differentiation Kit (Multi-purpose)( GUXMX-90021, cyagen, China), and OriCell^TM^ Mesenchymal Stem Cell Adipogenic Differentiation Medium (GUXMX-90031, cyagen, China) respectively according to the manufacturer's instructions.

### 2.8 Harvest and identification of senescent BMSC-EVs

Passage 2–4 BMSCs isolated from 20-month-old mice were cultured in high glucose DMEM medium with 10% EV-free FBS (C3801-0100, ViVaCell, China) for 48 ​h. Then the culture medium was collected and centrifuged at 3000 ​g for 30 ​min at 4 ​°C to remove dead cells and debris. Subsequently, the supernatant was centrifuged at 30,000 ​g for 1 ​h at 4 ​°C to remove big EVs. Next, EVs were isolated from the culture medium by centrifuging at 110,000 ​g for 2 ​h at 4 ​°C (CP100MX, Hitachi, Japan). The pellets were resuspended in phosphate buffer saline (PBS). The EVs were weighed by using the bicinchoninic acid (BCA) method.

### 2.9 Nanoparticle tracking analysis (NTA)

The size distribution and concentration of EVs were determined by NTA following the manufacturer's instructions. Briefly, the EVs resuspended and mixed in 30 ​μL PBS were injected into a particle size analyzer (N30E, NanoFCM, China), and the particle sizes were measured.

### 2.10 Transmission electron microscopy (TEM)

The isolated EVs were dropped onto a cupreous electron microscope grid and fixed with 10 ​μL uranyl acetate at RT for 1min. The samples were observed under a TEM (HT-7700, Hitachi, Japan) at 100 ​kV.

### 2.11 EVs labeling with Dil and EVs uptake assay

Purified EVs were labeled with the cell plasma membrane staining kit with Dil (C1991S, Beyotime, China). SCs were grown to 50% confluence in 12-well plates, and then the medium was replaced with high glucose DMEM containing 5 ​ng/mL Dil-labeled EVs. After incubation for 48 ​h, the SCs were fixed, and nuclei were stained with 4′, 6-diamidino-2-phenylindole (DAPI) (0100-20, SouthernBiotech, USA).

### 2.12 Treatment of BMSCs with small interfering RNA (siRNA) transfection against CD81

Lentivirus constructed CD81-specific siRNA (si-CD81) and control siRNA (si-NC) were purchased from Zolgene. BMSCs isolated from 20-month-old mice were seeded in 6-well plates. After BMSCs reached about 50% confluence, lentivirus constructed si-CD81 or si-NC at a multiplicity of infection (MOI) of 50% and 10 ​μg/mL polybrene (HB-PB-500, hanbio, China) were used to infect the BMSCs. The medium was changed 24 ​h after transfection.

### 2.13 Treatment of EVs

For *in vitro* EV treatment, 5 ​ng/mL EVs or an equal amount of PBS was added into the medium and replaced with each medium change. For *in vivo* EV treatment, 50 ​ng ​EV resuspended in 100 ​μL PBS was injected into the right side of the belly of the MG and TA of 4-month-old mice weekly for 4 weeks. An equal amount of PBS was injected into the left side of the belly of the MG and TA as control.

### 2.14 Administration of anti-CD81 antibodies (anti-CD81 Ab)

For blocking EVs *in vitro*, 10 ​μg/mL anti-CD81 Ab (A4863, Abclonal, China) [24] or an equal amount of PBS was incubated with the resuspended EVs for 2 ​h at RT. For *in vivo* anti-CD81 Ab treatment, 0.04 ​mg anti-CD81 Ab [25] resuspended in 0.5 ​mL normal saline (NS) was injected into the right side of the belly of the MG and TA of 20-month-old mice weekly for 4 weeks. An equal value of NS was injected into the left side of the belly of the MG and TA as control.

### 2.15 Western blotting analysis

Cells or EVs were sonicated into the lysis buffer supplemented with phosphatase and protease inhibitors (KGP2100, Keygen Biotech, China). Proteins were transferred onto PVDF membranes. Then the PVDF membranes were blocked by 5% milk (P0216-1500 ​g, Beyotime, China) and incubated with primary antibodies against GAPDH (1:10000, HRP-60004, proteintech, USA), P16 (1:1000, ab51243, abcam, UK), P53 (1:2000, A10610, ABclonal, China), P21 (1:2000, A1483, ABclonal, China), myogenic differentiation antigen (MyoD) (1:100, ab203383, abcam, UK), myogenin (MyoG) (1:200, ab124800, abcam, UK), myosin heavy chain (MyHC) (1:1000, ab91506, abcam, UK), CD9 (1:1000, ab263019, abcam, UK), CD81 (1:1000, ab109201, abcam, UK), TGS101 (1:1000, ab125011, abcam, UK), and Calnexin (1:1000, ab133615, abcam, UK) at 4 ​°C overnight. Membranes were then incubated with goat anti-rabbit IgG(H ​+ ​L) HRP (70-GAR0072, MultiSciences, China) at 37 ​°C for 1 ​h. Subsequently, the immune complexes were visualized using a tanon™ high-sig ECL western blotting substrate (180-5001, Tanon, China) and automatic digital gel/chemiluminescence image analysis system (4600SF, Tanon, China).

### 2.16 Biotin pull-down assay

Surface proteins of SCs were labelled with 2 ​mM EZ-Link Sulfo-NHS-LC-Biotin (A39257, Thermo scientific, USA) at RT for 30 ​min according to the manufacturer's instructions. Then proteins of EVs and biotin-labelled SCs were extracted with a cell lysis buffer for western and immune precipitation (P0013, Beyotime, China). The label of biotin was verified by western blotting using HRP-labeled streptavidin (1:10000, A0303, Beyotime, China). To perform the binding assay, 250 ​μL biotinylated surface proteins and 250 ​μL ​EV proteins were incubated for 4 ​h at 4 ​°C. Next, the mixed complex was incubated with streptavidin magpoly beads (SM01710, Smart-Lifesciences, China) for 30 ​min at RT. The beads were then washed three times and incubated with elution buffer for 5 ​min, followed by centrifugation. Eluted proteins were subjected to SDS–PAGE and visualized by coomassie blue staining (P0017F, Beyotime, China) or anti-CD81 antibody (anti-CD81 Ab) (1:500, ab109201, abcam, UK).

### 2.17 Hindlimb grip strength assessments

Hindlimb grip strength was measured by using a customized grip strength meter for mice (Taixing experimental instrument factory, China) before the mice were sacrificed. Hindlimb grip strength assessments were performed three times by the same individual. The maximum force (N) was selected.

### 2.18 Histology assessment

The muscle samples were fixed, embedded into paraffin, and sectioned. The sections were stained with Masson's trichrome staining (Keygen Masson's trichrome staining (KGMST)-8004, Keygen Biotech, China) according to the manufacturer's instructions. Quantification of fibrosis was determined by the area of blue staining and the cross-sectional area (CSA) was measured on 50 myofibres per sample.

### 2.19 Immunofluorescence analysis

The sections were blocked with quickblock blocking buffer for immune staining (P0260, Beyotime, China) for 15 ​min at RT, followed by incubation with primary antibody against Pax7 (1:100, bs-22741R, Bioss, China), MyoD (1:100, ab203383, abcam, UK), MyoG (1:500, ab124800, abcam, UK), fast MyHC (1:1000, ab91506, abcam, UK), laminin (1:50, ab11575, abcam, UK) at 4 ​°C overnight and labeled with Alexa Fluor594-preabsorbed goat anti-rabbit IgG (ab150084, Abcam, 1:500, UK), Alexa Fluor 488 AffiniPure F(ab')₂ Fragment Goat Anti-Rabbit IgG (111-546-003, Jackson ImmunoResearch, 1:500, USA) respectively for 2 ​h at room temperature. Next, the nucleus was stained with DAPI.

### 2.20 Micro-computed tomography (μCT)

Samples of femurs were dissected and scanned with high-resolution μCT (Inveon μPET-CT, Siemens, Germany) at a voltage of 80 ​kVp, 500 ​μA current and 15.0 ​μm resolutions per pixel. An inveon research workplace (version 4.2, Siemens, Germany) was used to perform 3D reconstruction analysis. Various parameters have been applied which included BMD, bone surface area/bone volume (BS/BV), bone surface area/total value (BS/TV), bone volume/total volume (BV/TV), trabecular number (Tb.N), trabecular thickness (Tb.Th).

### 2.21 Statistical analysis

All experiments were repeated three times, and the data were analysed using GraphPad Prism (9.0, Graph Software, USA). One-way analysis of variance (ANOVA) with Tukey's post-hoc test for multiple comparisons were applied to determine statistical significance between three groups, while student's t-tests were applied to determine statistical significance between two groups. We considered value of P ​< ​0.05 significant. The data was expressed as mean values and standard deviation (SD).

## 3 Results

### 3.1 Myogenic potential of SCs was significantly decreased with age

Since senescence involves a variety of biological behaviors, we first identified the most important biological behavior during senescence through bioinformatics analysis. With a |log2 FC| cutoff criteria >0.5 and P value ​< ​0.05, we identified 2566 upregulated DEGs and 2631 downregulated DEGs in aged SCs compared with young SCs in GSE47401 (Supplemental Fig. 1A and B). Then we performed GO functional enrichment analysis for the DEGs. We found that the DEGs were mainly involved in cell cycle phase transition, DNA replication, and negative regulation of cell cycle (Supplemental Fig. 1C). Meanwhile, 685 upregulated DEGs and 1005 downregulated DEGs in aged SCs compared with young SCs in GSE116585 were identified (Supplemental Fig. 1D and E). GO functional enrichment analysis indicated that the DEGs were mainly involved in mitotic nuclear division, chromosome segregation, and cell-substrate adhesion (Supplemental Fig. 1F). Among these enriched GO items, 844 GO items were enriched in both databases (Supplemental Fig. 1G). However, when we screened muscle-related items among these 844 GO items, we found that they were mainly involved in the development and differentiation rather than proliferation of muscle cells (Supplemental Fig. 1H and I). Similarly, by performing GSEA functional enrichment analysis, we found that there were 211 GSEA items that were both enriched in GSE47401 and GSE116585 (Supplemental Fig. 1J). Among these 211 GSEA items, only one GSEA item was related to muscle, namely muscle tissue development (Supplemental Fig. 1K and L).

So, we next tested the myogenic differentiation ability of senescent SCs *in vitro* and *in vivo*. SCs were isolated from quadriceps femoris, MG, and TA of 20-month-old mice with grip less than the upper quartile [26]. While SCs isolated from 4-month-old mice were used as young control (Supplemental Fig. 2A). The positive rate of β-galactosidase (β-gal) (Supplemental Fig. 2B and C) and the expression level of P16, P53, and P21 (Supplemental Fig. 2D–G) in senescent SCs were significantly higher than those in young SCs. Immunofluorescent staining (Supplemental Fig. 2H and I) and western blotting (Supplemental Fig. 2J–M) showed that expressions of two major myogenic markers, MyoD and MyoG, and the expression of MyHC was significantly decreased in senescent SCs compared with young SCs under myogenic induction.

As for *in vivo* test, the hindlimb grip strength of 20-month-old mice was significantly lower than that of 4-month-old mice (Supplemental Fig. 3A). HE and immunofluorescent staining further confirmed muscle atrophy of MG and TA in 20-month-old mice. HE staining showed that the CSA declined and the fibre area was increased in the MG (Supplemental Fig. 3B–E) and TA (Supplemental Fig. 3F–I) of 20-month-old mice. And immunofluorescent staining of fast MyHC was used to identify type I fibres and type II fibres. Immunofluorescent staining showed that CSA of type II fibres was significantly decreased in the MG (Supplemental Fig. 3J–L) and TA (Supplemental Fig. 3M−O) of 20-month-old mice. In addition, MyoD+ and MyoG+ ​myogenic SCs were also reduced in the MG (Supplemental Fig. 4A–D) and TA (Supplemental Fig. 4E–H) of 20-month-old mice compared with 4-month-old mice.

### 3.2 Senescent BMSCs-EVs impaired myogenesis of SCs

**Fig. 1:**
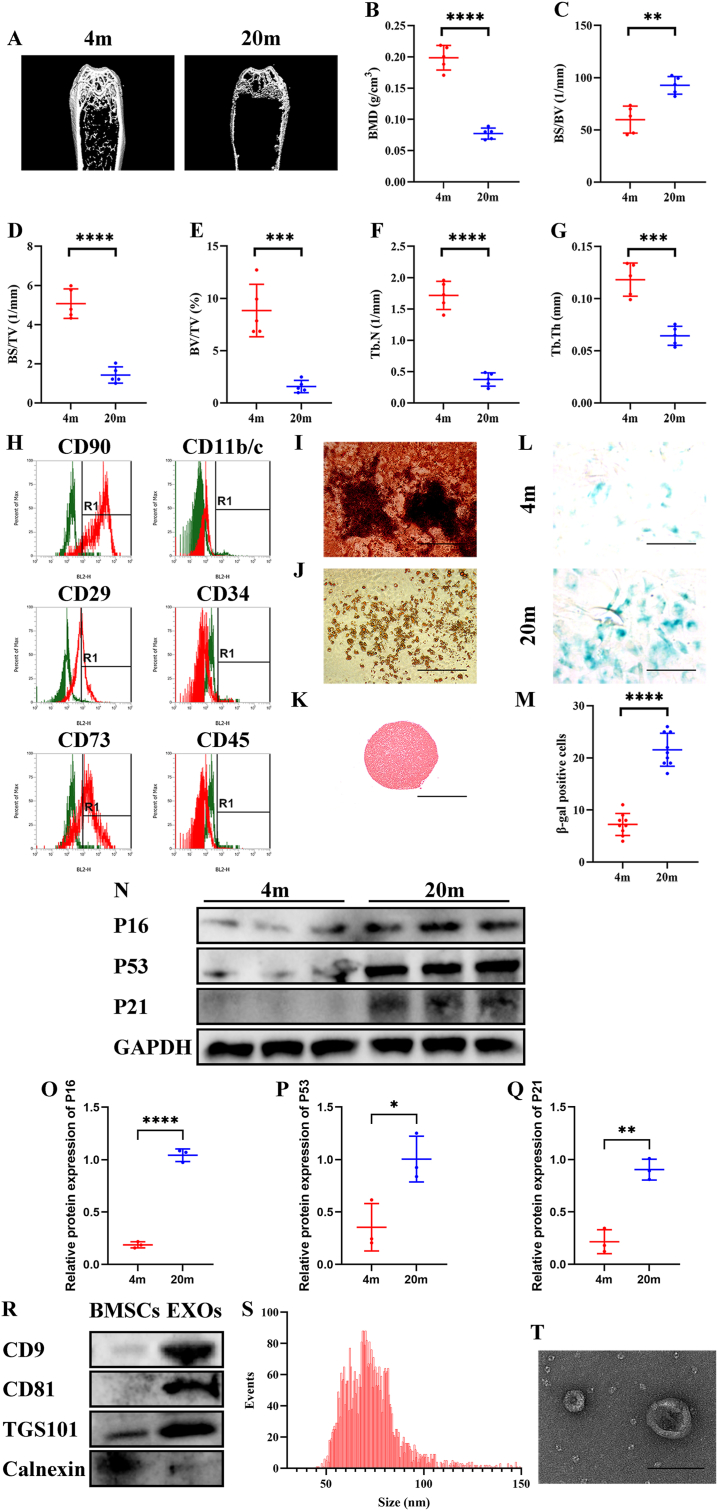
Harvest and identification of senescent BMSC-EVs from aged mice with osteoporosis. (A–G) Representative μ-CT (A) and quantitative analysis of BMD (B), BS/BV (C), BS/TV (D), BV/TV (E), Tb.N (F), and Tb.Th (G) of the femurs of 4-months-old and 20-months-old mice. (H) Flow cytometric assay of the surface marker of CD29, CD73, CD90, CD11, CD34 and CD45 on BMSCs. (I–K) Representative alizarin red O staining (I), oil red staining (J), Saf-O staining (K) of multilineage differentiation assay of BMSCs. (L and M) Representative β-gal staining (L) and β-gal positive cells counting (M). (N–Q) Western blotting (N) and quantitative analysis (O–Q) of P16, P53, and P21 level. GAPDH was used as a loading control. (R) Western blotting analysis of EV markers on BMSCs and BMSC-EVs. Calnexin was used as a negative control. (S) NTA of BMSC-EVs. (T) Representative TEM photograph of BMSC-EVs. (B–G) n ​= ​6. Values are shown as mean ​± ​SD. ∗∗P ​< ​0.01, ∗∗∗P ​< ​0.001, ∗∗∗∗P ​< ​0.0001, student's t-test. (M) n ​= ​3, three fields per sample were selected. Values are shown as mean ​± ​SD. ∗∗∗∗P ​< ​0.0001, student's t-test. (O–Q) n ​= ​3. Values are shown as mean ​± ​SD. ∗P ​< ​0.05, ∗∗P ​< ​0.01, ∗∗∗∗P ​< ​0.0001, student's t-test. (I–K) Scale bar ​= ​200 ​μm. (L) Scale bar ​= ​50 ​μm. (T) Scale bar ​= ​200 ​nm.

By using μCT, we confirmed that 20-month-old mice had osteoporosis with lower BMD, higher BS/BV, lower BS/TV, lower BV/TV, lower Tb.N, and lower Tb.Th compared to 4-month-old mice (Fig. 1A–G). The isolated BMSCs were positive for CD90, CD29, and CD73, and negative for CD11b/c, CD34, and CD45 (Fig. 1H), with the potential of multilineage differentiation (Fig. 1I–K). And BMSCs isolated from 20-month-old mice showed a higher positive rate of β-gal (Fig. 1L and M) and higher expression of P16, P53, and P21 (Fig. 1N–Q) compared to control. Next, western blotting showed that EV markers (CD9, CD81, and TSG101) were highly expressed in EVs, while these markers were expressed weakly in BMSCs (Fig. 1R). The NTA assay showed that the concentration of the BMSC-EVs was 7.71∗10^9 particles/mL and the size of the BMSC-EVs mainly distributed in 50–120 ​nm (Fig. 1S). And BMSC-EVs presented a spherical shape under TEM (Fig. 1T).

**Fig. 2:**
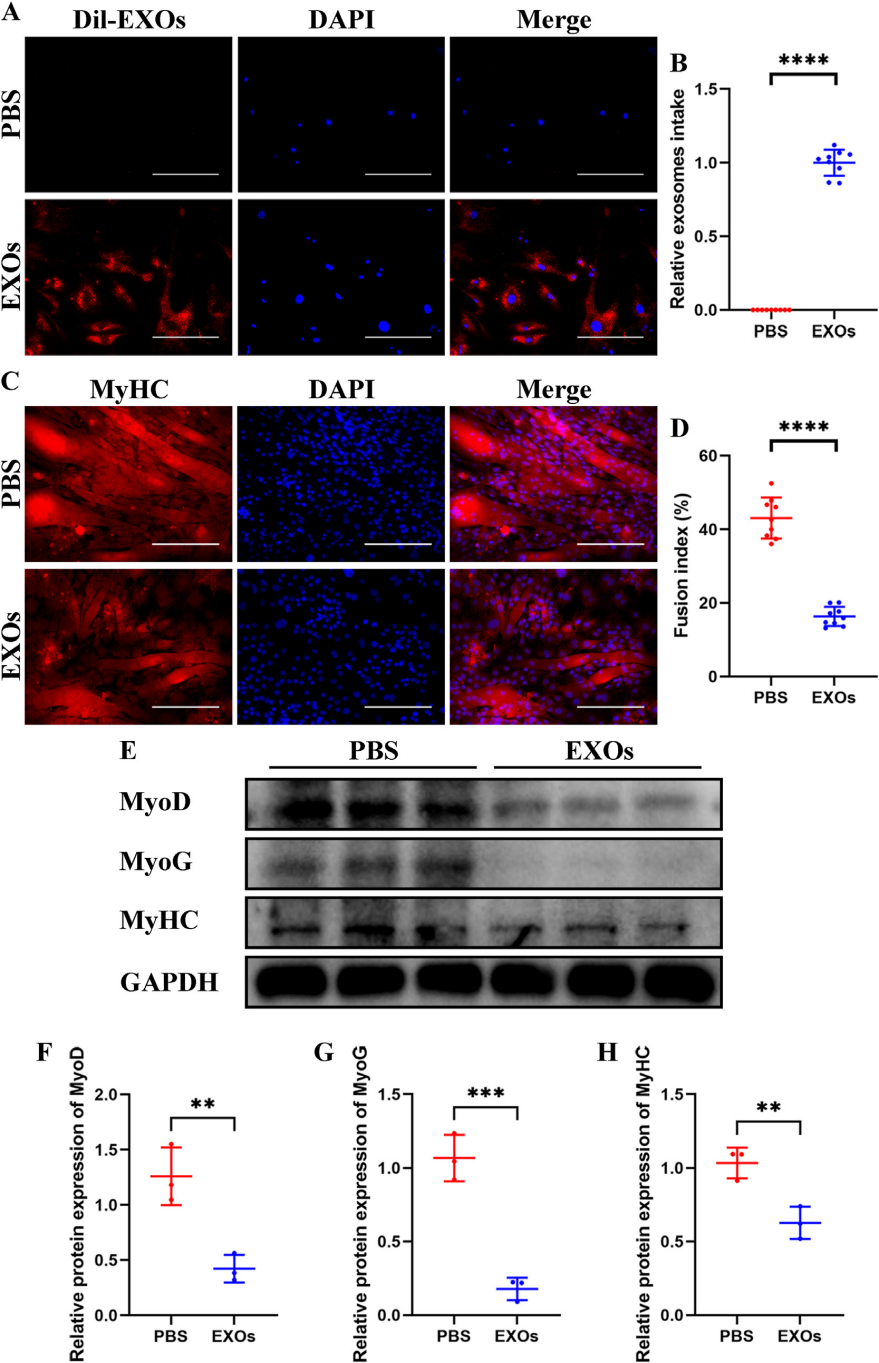
Senescent BMSC-EVs impaired myogenic differentiation of SCs *in vitro*. Young SCs were treated with senescent BMSC-EVs, and PBS treatment was used as negative control. (A and B) Representative fluorescence photographs of SCs treated with dil labeled BMSC-EVs (A) and quantitative analysis of relative intake of EVs (B). (C and D) Representative immunofluorescence staining of MyHC (C) and quantitative analysis of fusion index (D) of myogenic assay. (E–H) Western blotting (E) and quantitative analysis of MyoD (F), MyoG (G), and MyHC (H) levels during myogenic induction. GAPDH was used as a loading control. (B and D) n ​= ​3, three fields per sample were selected. Values are shown as mean ​± ​SD. ∗∗∗∗*P* ​< ​0.0001, student's t-test. (F–H) n ​= ​3. Values are shown as mean ​± ​SD. ∗∗*P* ​< ​0.01, ∗∗∗*P* ​< ​0.001, student's t-test. (A) Scale bar ​= ​50 ​μm. (C) Scale bar ​= ​200 ​μm.

Subsequently, young SCs were treated with senescent BMSC-EVs. Immunofluorescence assay showed that Dil labeled EVs were taken by SCs (Fig. 2A and B). And the myogenic potential of SCs was decreased after senescent BMSC-EVs treatment, presented as fewer formation of myotubes and lower fusion index (Fig. 2C and D) and lower expression of MyoD, MyoG, and MyHC (Fig. 2E–H).

**Fig. 3:**
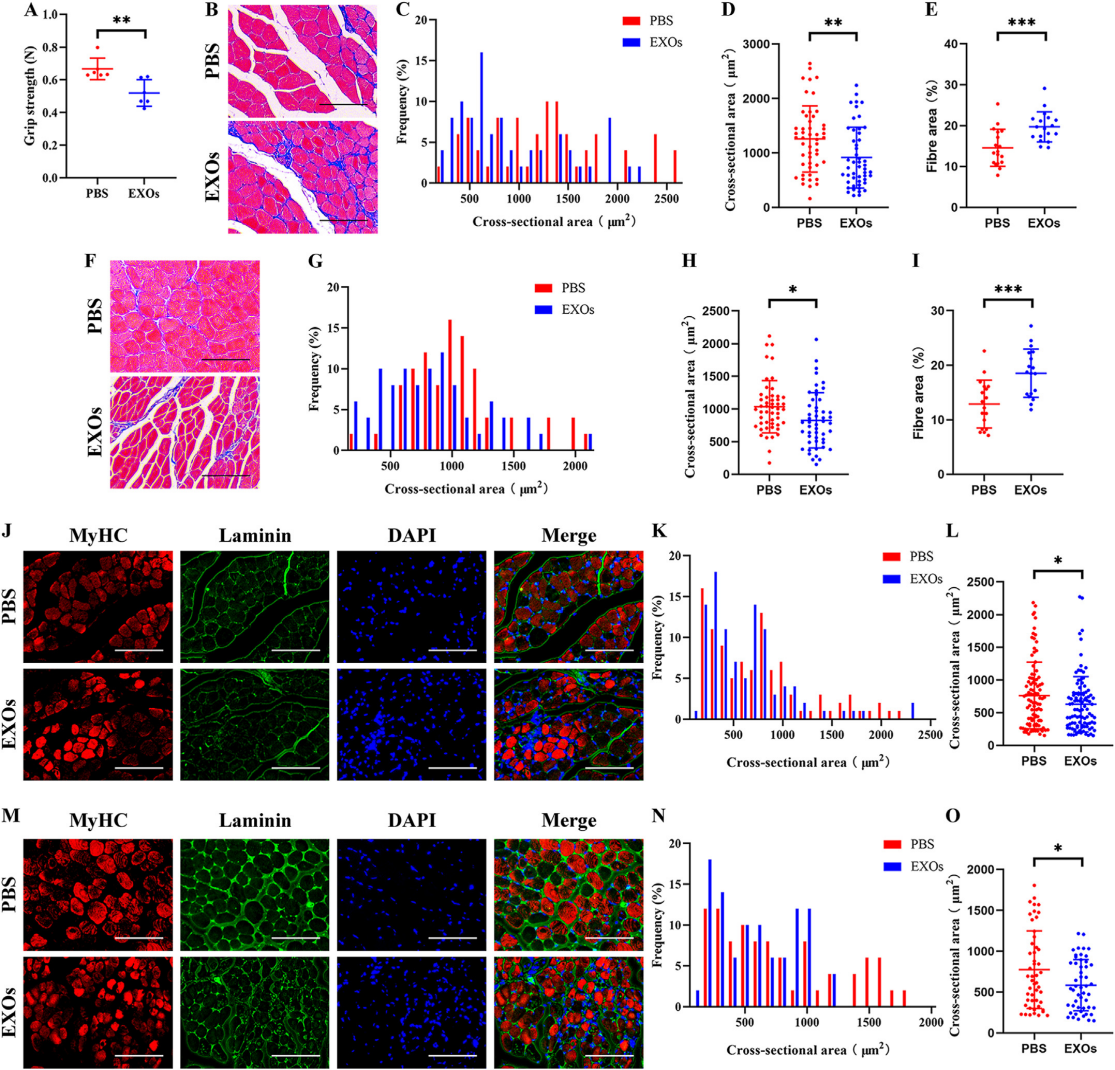
Local injection of senescent BMSC-EVs resulted in muscle atrophy in young mice. Senescent BMSC-EVs were locally injected into MG and TA of 4-month-old mice, and PBS treatment was used as negative control. (A) Hindlimb grip strength of 4-month-old mice was significantly decreased after senescent BMSC-EVs treatment. (B–E) Representative HE staining (B) and quantitative analysis of CSA (C and D) and fibre area (E) of MG. (F–I) Representative HE staining (F) and quantitative analysis of CSA (G and H) and fibre area (I) of TA. (J–L) Representative immunofluorescence staining of MyHC (J) and quantitative analysis of CSA of type II fibres (K and L) of MG. (M–O) Representative immunofluorescence staining of MyHC (M) and quantitative analysis of CSA of type II fibres (N and O) of TA. (A) n ​= ​6. Values are shown as mean ​± ​SD. ∗∗P ​< ​0.01, student's t-test. (C, D, G, H, K, L, N, and O) n ​= ​6, 50 myotubes were assessed. Values are shown as mean ​± ​SD. ∗∗P ​< ​0.01, ∗∗∗P ​< ​0.001, ∗∗∗∗P ​< ​0.0001, student's t-test. (E and I) n ​= ​6, three fields per sample were selected. Values are shown as mean ​± ​SD. ∗∗∗P ​< ​0.001, student's t-test. (B, F, J, and M) Scale bar ​= ​50 ​μm.

Hindlimb grip strength assessment indicated that senescent BMSC-EVs treatment impaired the hindlimb grip strength of 4-month-old mice (Fig. 3A). And HE staining demonstrated that local injection of senescent BMSC-EVs in MG (Fig. 3B–E) and TA (Fig. 3F–I) of the 4-month-old mice resulted in obvious muscle atrophy and fibrosis. Furthermore, we found that type II fibres in the MG (Fig. 3J-L) and TA (Fig. 3M−O) of the 4-month-old mice were atrophied presented as decreasing CSA.

### 3.3 Knockdown of CD81 in senescent BMSC-EVs attenuated impaired myogenesis of SCs induced by senescent BMSC-EVs

To evaluate whether CD81 protein on the surface of senescent BMSC-EVs mediated the intake of senescent BMSC-EVs by SCs, we first labeled biotin in the membrane proteins of SCs (Supplemental Fig. 5A). Subsequently, anti-CD81 primary antibody was used to incubate the PVDF membrane in biotin pulldown assay. We found that CD81 in the senescent BMSC-EVs could be bound to biotin-labeled membrane proteins of SCs, thus forming complexes with a molecular weight of about 110–130 kD and being pulldown (Supplemental Fig. 5B).

**Fig. 4:**
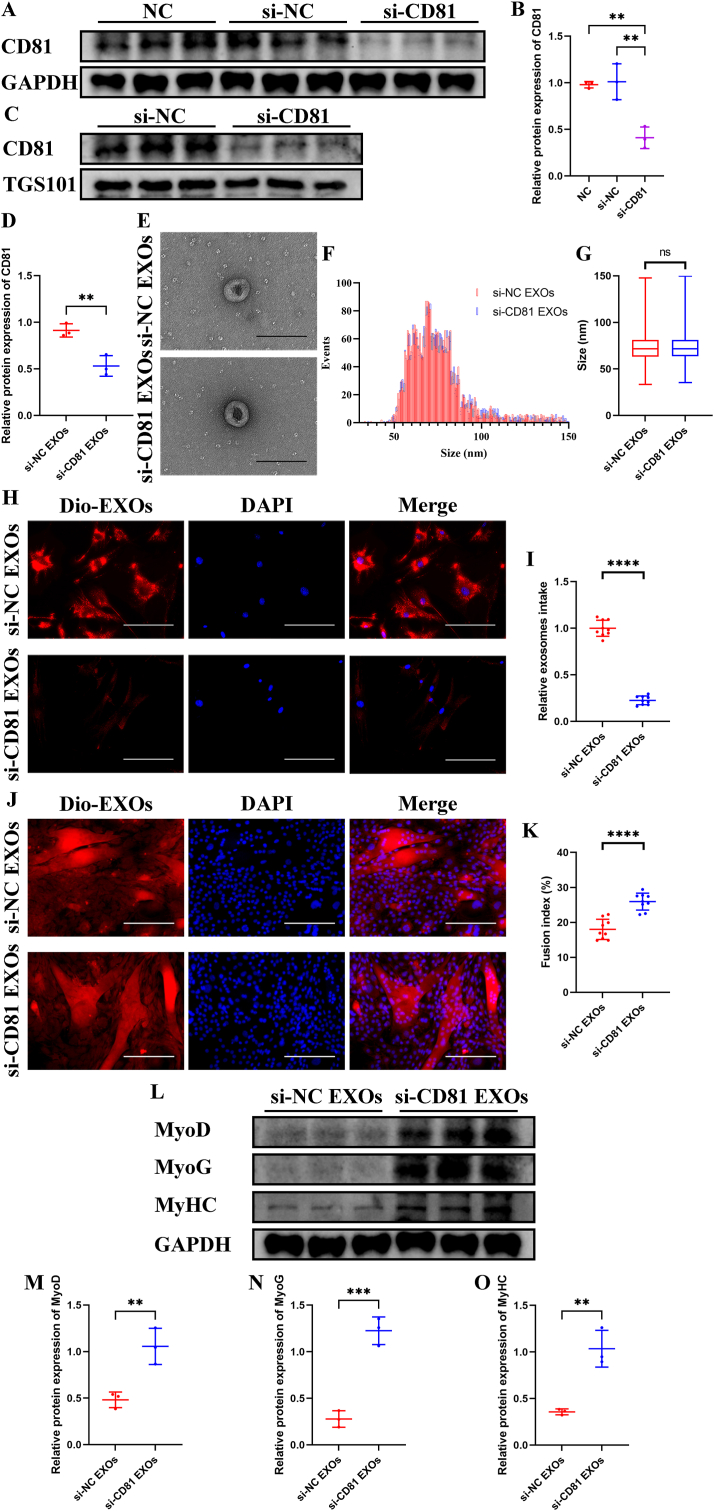
Knockdown of CD81 in BMSC-EVs prevented the harmful effects of senescent BMSC-EVs on myogenic differentiation of SCs *in vitro*. CD81 in senescent BMSCs were knockdowned by si-CD81. CD81-knockdown BMSC-EVs were harvested and used to treat young SCs. (A–D) Western blotting and quantitative analysis of CD81 level in CD81-knockdown BMSCs (A and B) and CD81-knockdown BMSC-EVs (C and D). GAPDH and TGS101 were used as loading controls. (E) Representative TEM photographs of si-NC and si-CD81 BMSC-EVs. (F and G) NTA (F) and quantitative analysis (G) of si-NC and si-CD81 BMSC-EVs. (H and I) Representative fluorescence photographs of SCs treated with dil labeled si-NC or si-CD81 BMSC-EVs (H) and quantitative analysis of relative intake of EVs (I). (J and K) Representative immunofluorescence staining of MyHC (J) and quantitative analysis of fusion index (K) of myogenic assay. (L–O) Western blotting (L) and quantitative analysis of MyoD (M), MyoG (N), and MyHC (O) levels during myogenic induction. GAPDH was used as a loading control. (B) n ​= ​3. Values are shown as mean ​± ​SD. ∗∗P ​< ​0.001, one-way ANOVA with Tukey's post-hoc test for multiple comparisons. (D, M–O) n ​= ​3. Values are shown as mean ​± ​SD. ∗∗P ​< ​0.001, ∗∗∗P ​< ​0.001, student's t-test. (G) Values are shown as mean ​± ​SD. Ns, no significance, student's t-test. (I and K) n ​= ​3, three fields per sample were selected. Values are shown as mean ​± ​SD. ∗∗∗P ​< ​0.001, ∗∗∗∗P ​< ​0.0001, student's t-test. (E) Scale bar ​= ​200 ​nm. (H) Scale bar ​= ​50 ​μm. (J) Scale bar ​= ​200 ​μm.

Then we used si-CD81 constructed in lentivirus to silence CD81 in senescent BMSCs. Western blotting showed that CD81 lowly expressed in BMSCs transfected with si-CD81 compared with BMSCs transfected with si-NC (Fig. 4A and B). The EVs collected from CD81-silenced BMSCs also presented lower expression of CD81 when normalized to the expression level of TGS101 (Fig. 4C and D). Both si-CD81 EVs and si-NC EVs maintained spherical shape and their diameter maintained 30–150 ​nm without a significant difference in particle size (Fig. 4E–G). Knockdown of CD81 significantly inhibited uptake of senescent BMSC-EVs by young SCs (Fig. 4H and I), so the negative effects of senescent BMSCs in myogenesis of SCs were attenuated. The myotube formation was increased in si-CD81 EVs treated SCs compared with those treated with si-NC EVs (Fig. 4J and K). And the expressions of MyoD, MyoG, and MyHC were also higher in SCs treated with si-CD81 EVs than those in the control group (Fig. 4L-O).

**Fig. 5:**
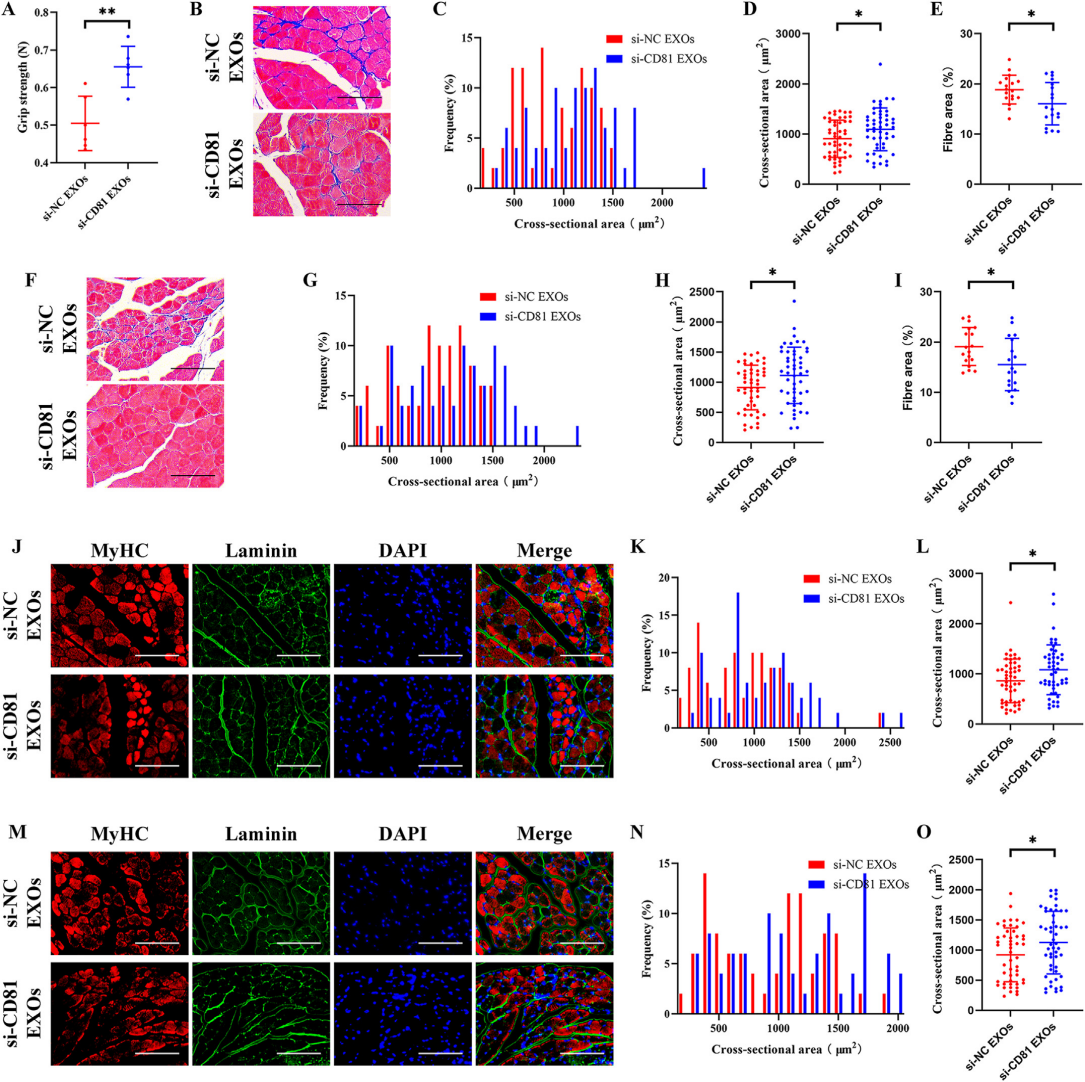
Knockdown of CD81 in BMSC-EVs prevented the effects of senescent BMSC-EVs on promoting muscle atrophy. 4-month-old mice were subject to local injection of si-NC or si-CD81 BMSC-EVs in MG and TA. (A) Hindlimb grip strength of 4-month-old mice was effectively maintained in the si-CD81 EVs treatment group. (B–E) Representative HE staining (B) and quantitative analysis of CSA (C and D) and fibre area (E) of MG. (F–I) Representative HE staining (F) and quantitative analysis of CSA (G and H) and fibre area (I) of TA. (J–L) Representative immunofluorescence staining of MyHC (J) and quantitative analysis of CSA of type II fibres (K and L) of MG. (M–O) Representative immunofluorescence staining of MyHC (M) and quantitative analysis of CSA of type II fibres (N and O) of TA. (A) n ​= ​6. Values are shown as mean ​± ​SD. ∗∗P ​< ​0.01, student's t-test. (C, D, G, H, K, L, N, and O) n ​= ​6, 50 myotubes were assessed. Values are shown as mean ​± ​SD. ∗P ​< ​0.05. (E and I) n ​= ​6, three fields per sample were selected. Values are shown as mean ​± ​SD. ∗P ​< ​0.05, student's t-test. (B, F, J, and M) Scale bar ​= ​50 ​μm.

And we found that si-CD81 EVs treatment group got a higher hindlimb grip strength (Fig. 5A). Compared with the si-NC EVs treatment group, MG (Fig. 5B–E) and TA (Fig. 5F–I) of the 4-month-old mice subjected to local injection of si-CD81 EVs maintained higher CSA and lower level of fibrosis. In addition, as showed in immunofluorescence staining, the CSA of type II fibres in MG (Fig. 5J-L) and TA (Fig. 5M-G) in the si-CD81 treatment group was all maintained at a higher level compared with the si-NC EVs treatment group.

### 3.4 Antibody blocking of CD81 on the surface of senescent BMSC-EVs partly attenuated sarcopenia in aged mice

**Fig. 6:**
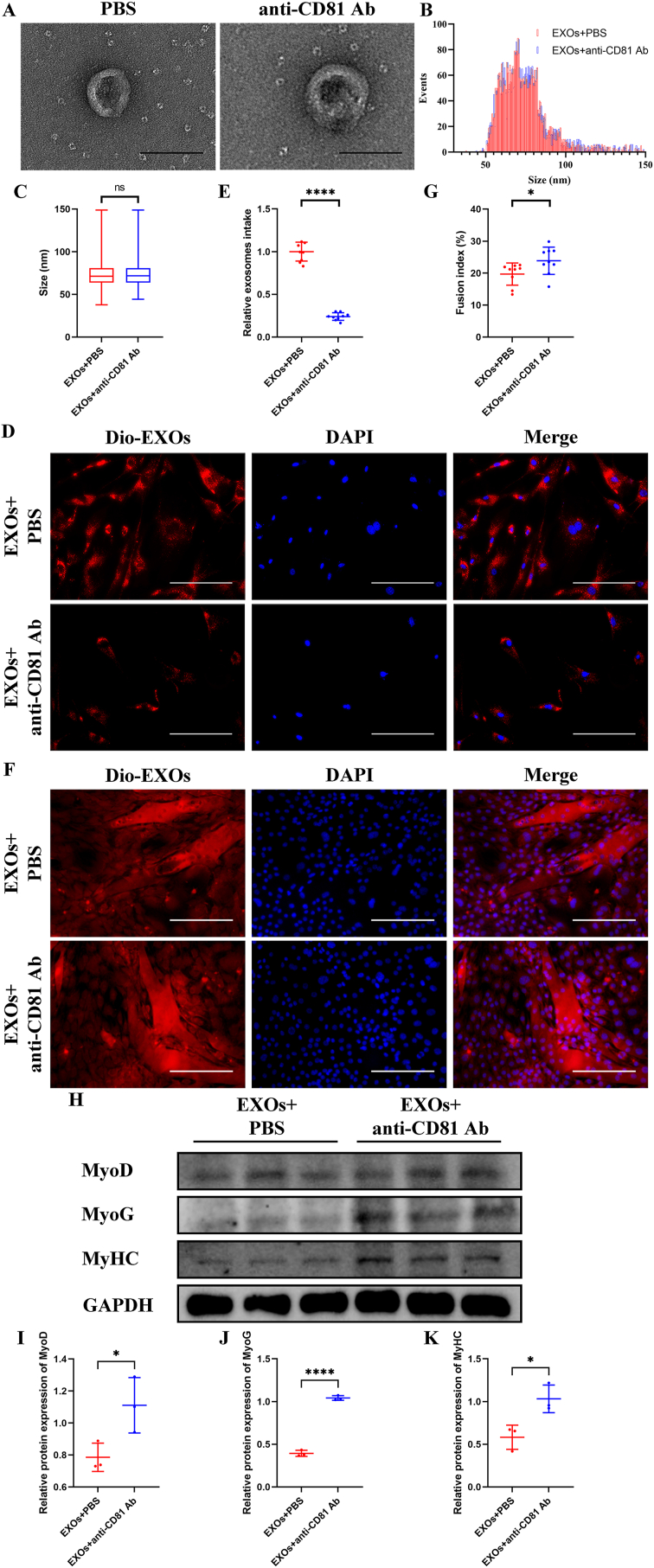
Blocking CD81 on the surface of senescent BMSC-EVs prevented the harmful effects of senescent BMSC-EVs on myogenic differentiation of SCs *in vitro*. Senescent BMSC-EVs were blocked with anti-CD81 Ab and then incubated with young SCs. (A) Representative TEM photographs of senescent BMSC-EVs with or without anti-CD81 incubation. (B and C) NTA (B) and quantitative analysis (C) of senescent BMSC-EVs with or without anti-CD81 incubation. (D and E) Representative fluorescence photographs of SCs treated with dil-labeled anti-CD81-Ab-incubated BMSC-EVs (D) and quantitative analysis of relative intake of EVs (E). (F and G) Representative immunofluorescence staining of MyHC (F) and quantitative analysis of fusion index (G) of myogenic assay. (H–K) Western blotting (H) and quantitative analysis of MyoD (I), MyoG (J), and MyHC (K) levels during myogenic induction. GAPDH was used as a loading control. (C) Values are shown as mean ​± ​SD. Ns, no significance, student's t-test. (E and G) n ​= ​3, three fields per sample were selected. Values are shown as mean ​± ​SD. ∗∗P ​< ​0.01, ∗∗∗∗P ​< ​0.0001, student's t-test. (I–K) n ​= ​3. Values are shown as mean ​± ​SD. ∗P ​< ​0.05, ∗∗∗∗P ​< ​0.0001, student's t-test. (A) Scale bar ​= ​200 ​nm. (D) Scale bar ​= ​50 ​μm. (F) Scale bar ​= ​200 ​μm.

Though silencing CD81 in senescent BMSC-EVs achieved desirable results, knockdown of CD81 *in vivo* remained difficult to apply clinically. So, we aimed to use anti-CD81 Ab to block CD81 on the surface of senescent BMSC-EVs to prevent the uptake of senescent BMSC-EVs by SCs. By using TEM and NTA assay, we found that anti-CD81 Ab blocking did not affect the particle size and morphology of BMSC-EVs (Fig. 6A–C). Uptake of senescent BMSC-EVs by young SCs was effectively inhibited after anti-CD81 Ab blocking (Fig. 6B and C). So that the harmful effect of senescent BMSC-EVs on the myogenesis of SCs was significantly alleviated (Fig. 6D and E). Also, the expressions of MyoD, MyoG, and MyHC were maintained in the SCs treated with senescent BMSC-EVs blocked by anti-CD81 Ab during myogenic induction compared with the simple senescent BMSC-EVs treatment group (Fig. 6F–I).

**Fig. 7:**
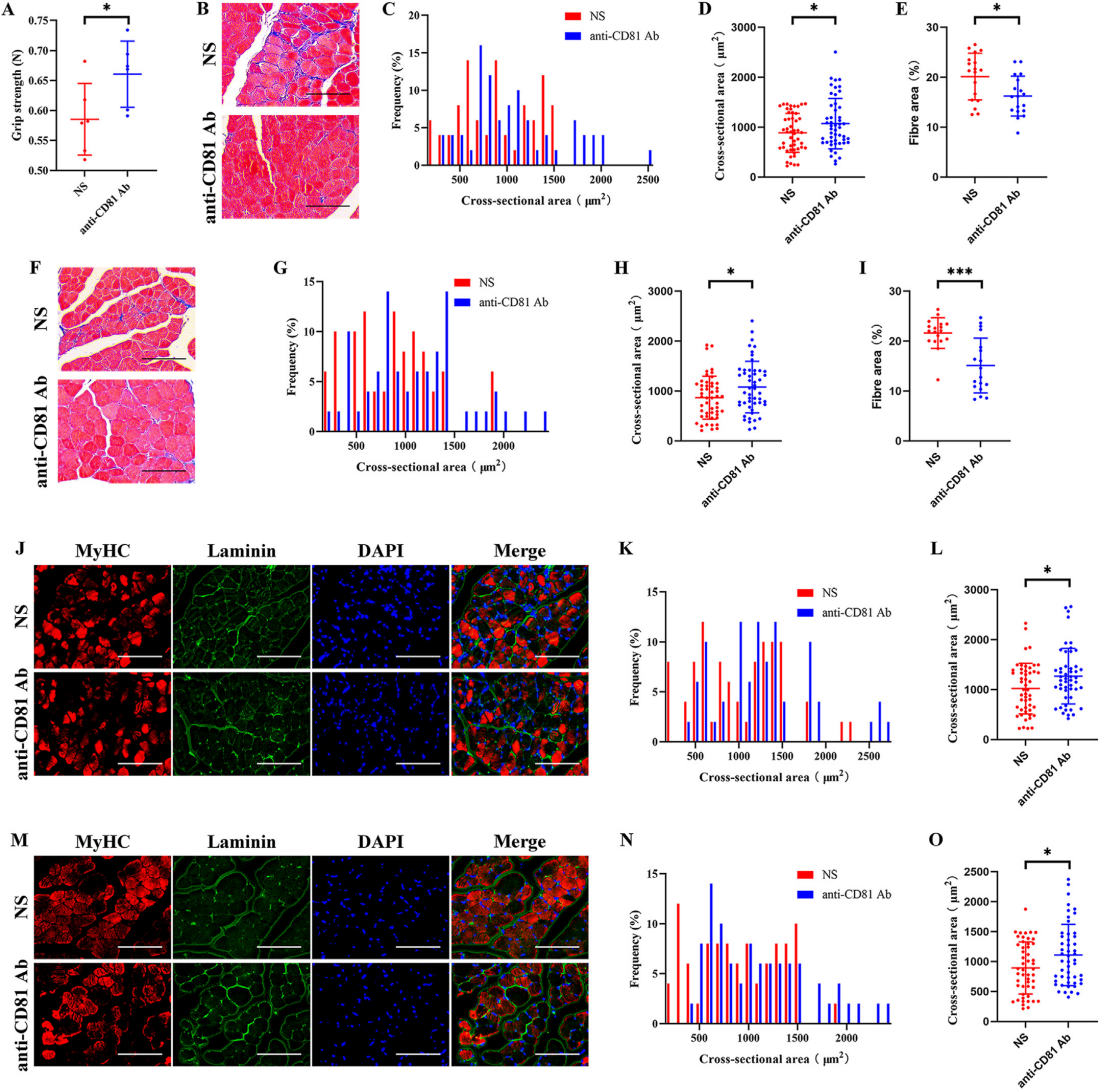
Local injection of anti-CD81 Ab attenuated sarcopenia in aged mice. 20-month-old mice were subject to local injection of anti-CD81 Ab in MG and TA, and PBS treatment was used as negative control. (A) Hindlimb grip strength of 20-month-old mice was increased after anti-CD81 Ab treatment. (B–E) Representative HE staining (B) and quantitative analysis of CSA (C and D) and fibre area (E) of MG. (F–I) Representative HE staining (F) and quantitative analysis of CSA (G and H) and fibre area (I) of TA. (J–L) Representative immunofluorescence staining of MyHC (J) and quantitative analysis of CSA of type II fibres (K and L) of MG. (M–O) Representative immunofluorescence staining of MyHC (M) and quantitative analysis of CSA of type II fibres (N and O) of TA. (A) n ​= ​6. Values are shown as mean ​± ​SD. ∗P ​< ​0.05, student's t-test. (C, D, G, H, K, L, N, and O) n ​= ​6, 50 myotubes were assessed. Values are shown as mean ​± ​SD. ∗P ​< ​0.05. (E and I) n ​= ​6, three fields per sample were selected. Values are shown as mean ​± ​SD. ∗P ​< ​0.05, ∗∗∗P ​< ​0.001, student's t-test. (B, F, J, and M) Scale bar ​= ​50 ​μm.

And the hindlimb grip strength decline, muscle atrophy and fibrosis of MG and TA (Fig. 7A–I) in 20-month-old mice was attenuated after local injection of anti-CD81 Ab. In addition, the CSA of type II fibres of MG (Fig. 7J–L) and TA (Fig. 7M−O) of 20-month-old mice treated with locally injection of anti-CD81 Ab maintained at a higher level.

## 4 Discussion

Skeletal muscle mass and function decline with age, which leads to frailty [27]. Sarcopenia increases the risk of falls and fractures in patients with osteoporosis [2]. As two major age-related musculoskeletal diseases, osteoporosis and sarcopenia are partly caused by declined function of BMSCs and SCs, respectively [7,10,11]. And, increased incidence of sarcopenia is observed in patients with osteoporosis [4,5]. Here, we aim to reveal the effect of senescent BMSCs on SCs, and explore an approach to prevent sarcopenia in patients with osteoporosis. To date, there are several mouse models of osteoporosis and sarcopenia, including naturally aged, accelerated senescent, disuse, and ovariectomized mouse model [[28], [29], [30]]. Naturally aged animal models have advantages over other animal models because that similar co-morbidities found in human osteoporosis and sarcopenia patients could be expected. And it has been verified that C57BL/6J mice over 20 months of age have obvious osteoporosis [31] and sarcopenia [32], presenting as significant decline in bone mass, muscle mass, and grip strength. Thus, we chose to use naturally aged C57BL/6J mice in the present study.

It is well recognized that muscle regeneration potentials of SCs are reduced with aging, thus leading to muscle atrophy [33]. Muscle regeneration involves multiple biological behaviour. But which biological behaviour would be most important remained unclear. Our results of bioinformatics analysis of young and aged SCs indicate that DEGs are mainly enriched in muscle cell differentiation. Equivocal results were found in comparing the number of SCs between young and old individuals. Sousa-Victor et al. and Day et al. reported that the number of SCs was decreased in old muscle [34,35]. But Brzeszczyńska et al. found that both PAX3 and PAX7 were high expressed in elderly donors [36]. And Brooks et al. demonstrated that the number of SCs did not change in aged muscle [37]. The reason for these results may be that the self-renew potential of SCs varies between individuals. In addition, Sousa-Victor et al. found that in C57/BL6 mice, the decline of activation and proliferation of SCs was prominent after 28 months, while muscle atrophy was detected at 20–24 months. Furthermore, it has been reported that depleting SCs in adult and aged mice did not affect sarcopenia [38]. Therefore, alterations in the ability of SCs to proliferate and self-renew in elderly individuals may not be the main cause of sarcopenia. On the contrary, several literature conformably states that the potential for myogenic differentiation of SCs declined with aging [34,36,39]. We also found that old SCs in 20-month-old mice exhibited lower myogenic differentiation *in vitro* and *in vivo* compared with young control.

Previous studies have reported that cargos in EVs, such as miRNAs and proteins, change during the aging process. And these changes in EV cargos play important roles in crosstalk between cells and tissues, thus transmitting age-related stimuli to target cells and tissues [9,39]. Senescent C2C12 ​cells released EVs into circulation and induced senescence of BMSCs [40]. And our work indicates that EVs transmit from senescent BMSCs to young SCs, thus impairing myogenesis of young SCs. We used young SCs and young mice in EVs treatment assay because that it is young SCs remained in muscle rather than old SCs would play a major role in muscle regeneration, since Baker et al. found that eliminating senescent cells improved mass and exercise ability of skeletal muscle [41]. Che et al. demonstrated that EVs and CM derived from passage 10 BMSCs promoted myogenic differentiation of C2C12 ​cells via the AKT/mTOR pathway [8]. These differences may be due to the following reasons. First, different from EVs, CM contains additional contents, such as growth factors and inflammatory factors. Xu et al. reported that EVs and CM derived from vascular smooth muscle cells (VSMCs) played distinct roles in regulating senescence and osteogenesis of VSMCs [42]. Second, there are some differences between the natural senescence *in vivo* and the replicative senescence *in vitro* [43]. The degree of senescence of passage 10 BMSCs may not be as much as the senescent BMSCs in aged mice. In support of this view, Che et al. also found that CM derived from passage 15 BMSCs damaged myogenic differentiation of C2C12 ​cells [8].

Then we hope to find an approach to block the uptake of senescent BMSC-EVs by SCs. Selection and uptake of EVs by target cells are mediated by complexes on the membranes of EVs [15]. CD81 is a tetraspanin that abundant on the membranes of EVs and be considered as a marker of EVs [44]. And it has been confirmed that CD81 can interact with integrins to form complexes [16,45]. Complexes comprised of CD81 and CD29 on the membranes of target cells play an important role in EV uptake by recipient cells [46]. In the present study, biotin pulldown assay showed that CD81 could interact with the biotin-labeled membrane proteins, thus being pulled down. The band of pulldown CD81 was located in approximately 110–140 kD. Molecular weight of CD81 is 26 kD. Spliced CD81 is also detected at the molecular weight of 20 kD [47]. Molecular weights of integrin α5 and β1 is 115 kD and 88 kD, respectively. And the spliceosome of integrin β1 is detected at the molecular weight of 115 kD and 135 kD [48]. Therefore, depending on the molecular weight of the pulldown complex, this complex may be composed of CD81 and integrins.

To verify our hypothesis, we next knocked down CD81 in senescent BMSCs by using lentivirus constructed si-CD81. As expected, compared to the si-NC EVs, fewer si-CD81 EVs were internalized by SCs. And si-CD81 EVs showed milder damage to the myogenic differentiation of SCs. Subsequently, we hoped to apply reagents rather than lentivirus to inhibit the function of CD81 on EV uptake. It has been reported that anti-CD81 Ab could effectively block CD81 on the membrane of recipient cells [24,25,49]. So, we tried to use anti-CD81 Ab to block CD81 on senescent BMSC-EVs. After anti-CD81 Ab incubation, the uptake of senescent BMSC-EVs by SCs was significantly decreased thus relieving the damage of senescent BMSC-EVs on myogenesis of SCs. Fortunately, we found that anti-CD81 Ab administration effectively attenuated muscle mass in elderly mice. Interestingly, Tachibana et al. found that 10 ​μg/mL anti-CD81 Ab markedly delayed muscle cell fusion and myotube formation, but did not influence the expression of MyHC of C2C12 ​cells during myogenic induction *in vitro* [45]. However, in the present study, anti-CD81 Ab was used to incubate with BMSC-EVs rather than SCs, so that anti-CD81 Ab did not interact with SCs directly. So we got an opposite result in the present study. In addition, anti-CD81 Ab can also regulate the motility and fusion of macrophages and the expression of metalloproteinase in macrophages [50,51]. Healthy macrophages enhance muscle regeneration of SCs [32,52]. While aged macrophages impair proliferation, fusion, and myogenesis of SCs, which leads to sarcopenia [[53], [54], [55]]. Thus, the treatment effect of anti-CD81 Ab on sarcopenia *in vivo* might be partly due to that anti-CD81 Ab influenced the macrophages in the muscle.

EVs have been verified to be important intercellular and inter-organization communicators as EVs can transfer mRNAs, non-coding RNAs, and proteins to target cells, thus mediating the functions of target cells [44]. However, apart from benefit effects, MSC-EVs can also have harmful effects on target cells, such as aggravating kidney injury [56], trigging heart disease [57], and promoting tumor [58], as well as aggravating muscle atrophy. Thus, we speculate that blocking CD81 on the surface of EVs could also be a potential approach to attenuate the harmful effects of MSC-EVs on other target cells. In addition, it has been reported that the EV selection and uptake by target cells can be modulated by expressing defined tetraspanin on the EVs [15]. So, CD81-over-expressed EVs have potential to serve as candidates for drug delivery targeting SCs.

There are still some limitations in the present study. First, our results were descriptive and relatively primary. The mechanism by which senescent BMSC-EVs impair the myogenic differentiation of SCs remains unclear. Second, anti-CD81 Ab may have multi-target effects *in vivo*. Among these effects, whether the promotion of myogenic differentiation of SCs is the most important also needs to be further verified.

## 5 Conclusions

**Fig. 8:**
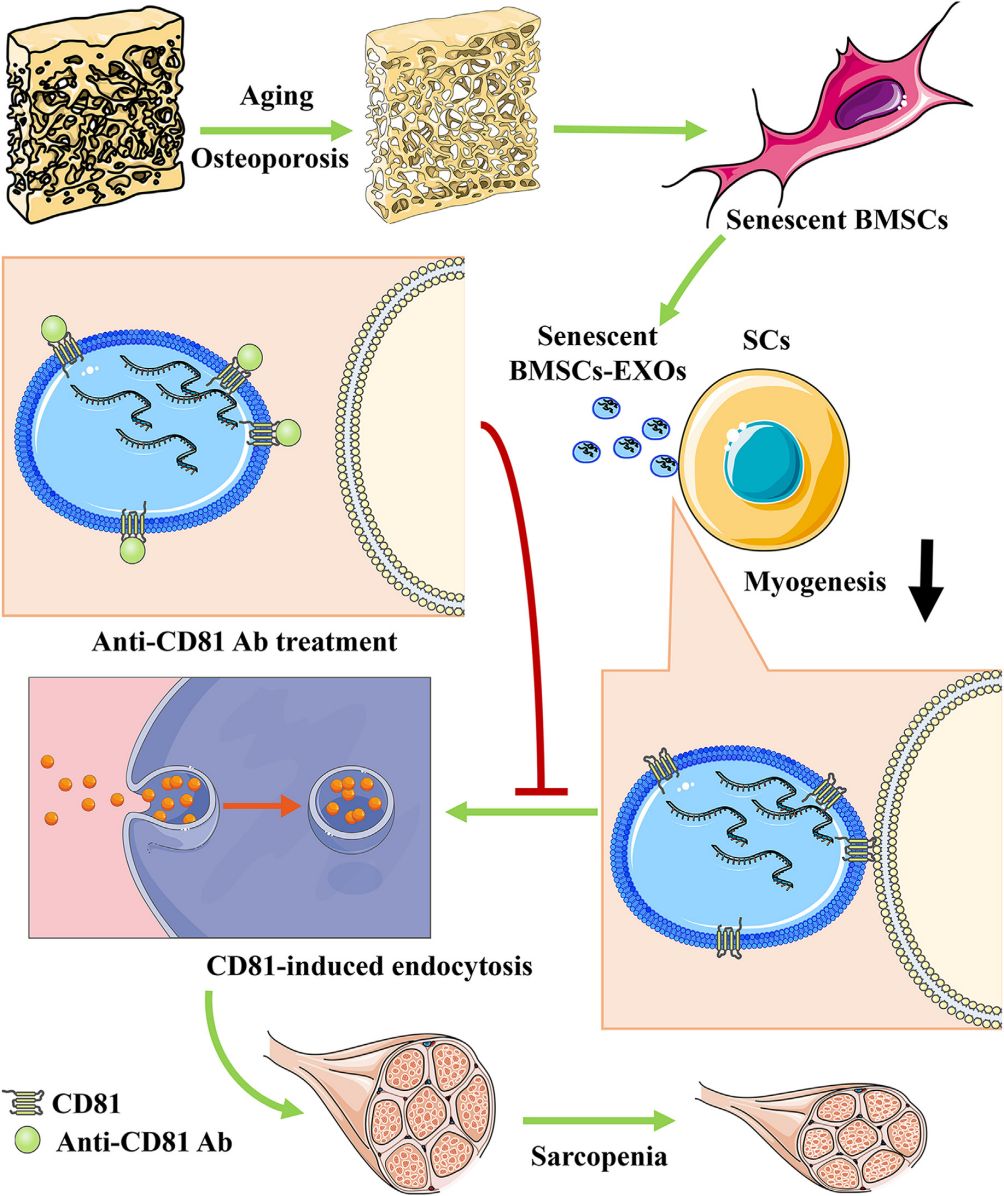
Senescent BMSC-EVs play a negative role on the myogenic differentiation of SCs. CD81 on the surface of senescent BMSC-EVs mediated the EV uptake by SCs. Blocking CD81 on the surface of senescent BMSC-EVs by using anti-CD81 antibody could attenuate muscle atrophy.

In conclusion, this study demonstrated that senescent BMSC-EVs play a negative role in the myogenic differentiation of SCs. CD81 on the surface of senescent BMSC-EVs mediated the EV uptake by SCs. And blocking CD81 on the surface of senescent BMSC-EVs could attenuate muscle atrophy in aged mice (Fig. 8), which could be useful for prevention and treatment of sarcopenia in patients with osteoporosis in clinical practice.

## Author contribution

Hanhao Dai: Conceptualization, Methodology, Investigation, Data Curation, Writing-Original Draft, Funding acquisition. Wu Zheng: Investigation, Visualization, Funding acquisition. Jun Luo: Investigation, Data Curation, Funding acquisition. Guoyu Yu: Data Curation. Chao Song: Visualization. Yijing Wu: Writing-Review & Editing. Jie Xu: Conceptualization, Methodology, Writing-Review & Editing, Funding acquisition. All the authors approved the manuscript submission.

## Funding

This work was supported by 10.13039/501100003392Fujian Provincial Natural Science Foundation Projects (2021J01376), Joint Project for Health and Education of Fujian Province (2019-WJ-01), Health Research Personnel Training Project of 10.13039/501100014125Fujian Provincial Health Commission (2019-CX-1), Science and Technology Planning Project of Fujian Province (2019J01173), 10.13039/501100017543Fujian Provincial Hospital Firestone Fund (2019024HSJJ), 10.13039/501100017543Fujian Provincial Hospital Firestone Fund (2020029HSJJ), and Startup Fund for Scientific Research of 10.13039/501100013795Fujian Medical University (2020QH2050).

## Data availability statements

The datasets used and/or analyzed during the current study are available from the corresponding author on reasonable request.

## Ethics approval and consent to participate

All animal experiments were approved by the Animal Ethics Committee of Fujian Medical University (NO. 2019-0134).

## Consent for publication

Consent for publication was obtained from all authors.

## Declaration of competing interest

All the authors declare no conflicts of interest with the contents of this article.
