# Supplementary material for: Inhibiting uptake of extracellular vesicles derived from senescent bone marrow mesenchymal stem cells by muscle satellite cells attenuates sarcopenia
Source: J Orthop Translat. 2022 Jul 6;35:23–36. doi: 10.1016/j.jot.2022.06.002 (PMC9260455; doi:10.1016/j.jot.2022.06.002)
Supplement: Multimedia component 1 [file mmc1.docx]

**
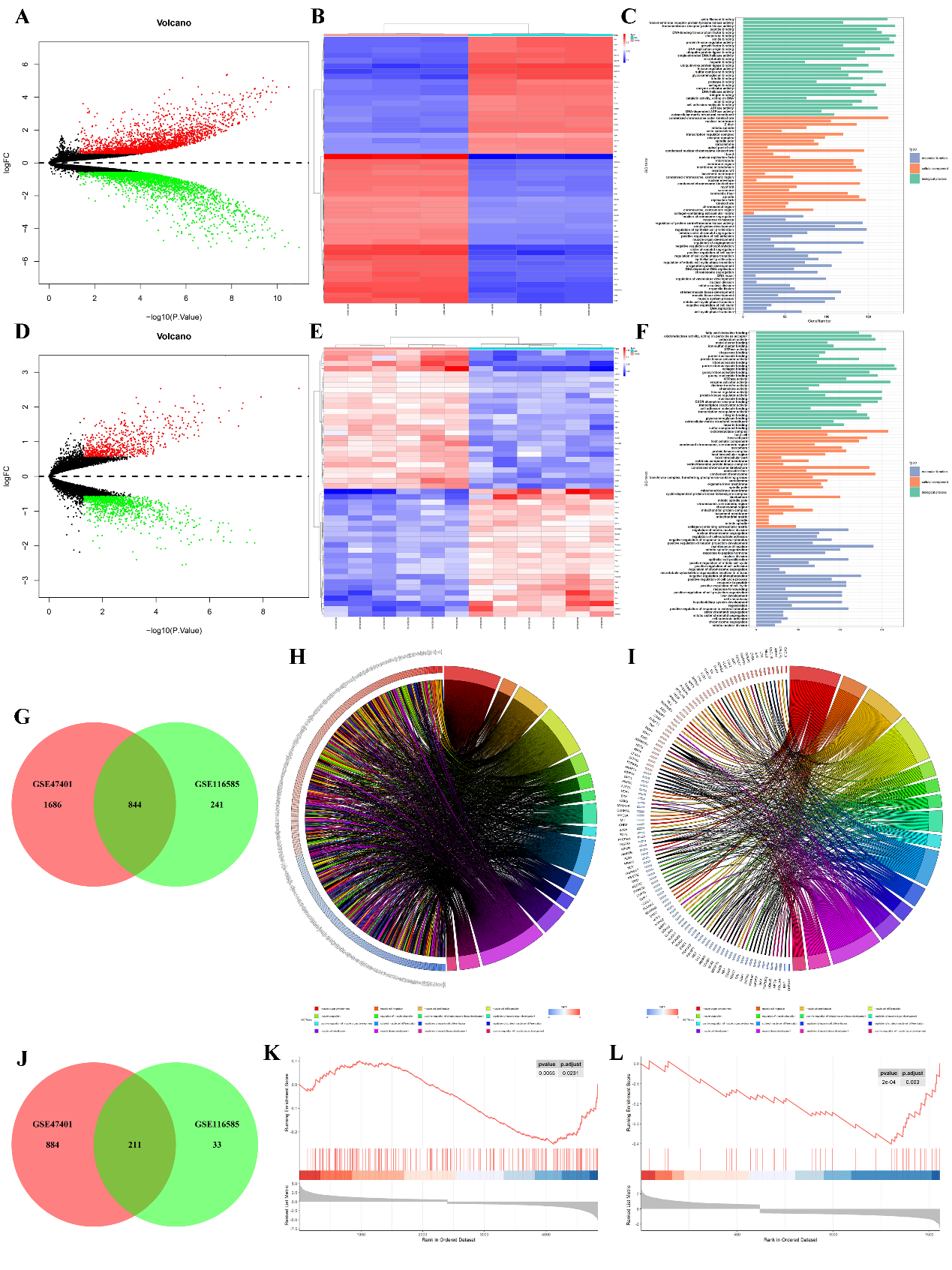
**

**Supplemental Fig. 1. DEGs in senescent SCs were mainly enriched in muscle cell differentiation.** (A and B) Volcano plots (A) and hierarchical clustering heatmap (B) of DEGs in GSE47401. C, Enrichment of top 30 GO terms of DEGs in GSE47401. (D and E) Volcano plots (D) and hierarchical clustering heatmap (E) of DEGs in GSE116585. (F) Enrichment of top 30 GO terms of DEGs in GSE116585. (G) Venn diagram of common GO terms that both enriched in GSE47401 and GSE116585. (H) Chord diagram of the DEGs according to the common GO terms involved in muscle development in GSE47401. (I) Chord diagram of the DEGs according to the common GO terms involved in muscle development in GSE116585. (J) Venn diagram of common GSEA terms that both enriched in GSE47401 and GSE116585. (K) GSEA enrichment plot of DEGs related to muscle cell differentiation in GSE47401. (L) GSEA enrichment plot of DEGs related to muscle cell differentiation in GSE116585.


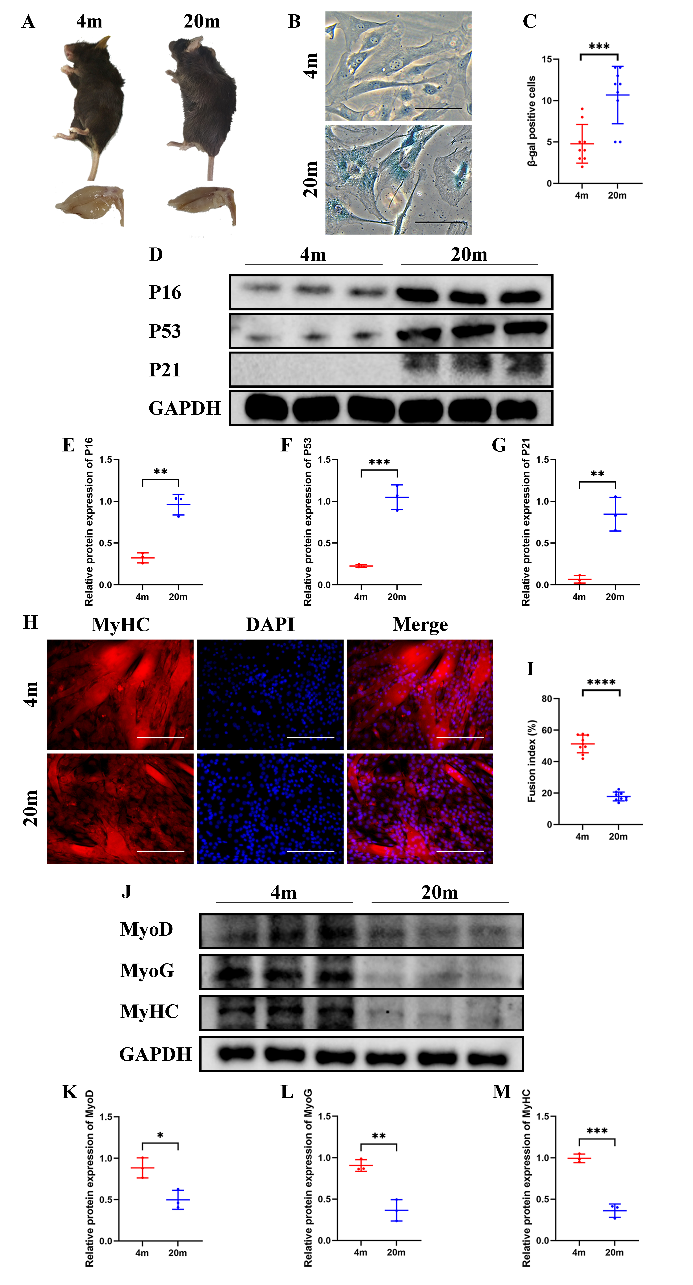


**Supplemental Fig. 2. Myogenic potential of isolated senescent SCs was significantly decreased. (**A) Compared with 4-minths-old mice, 20-months-old mice showed obviously frail with muscle atrophy. (B and C) Representative β-gal staining (B) and β-gal positive cells counting (C). (D-G) Western blotting (D) and quantitative analysis (E-G) of P16, P53, and P21 level. GAPDH was used as a loading control. (H and I) Representative immunofluorescence staining of MyHC (H) and quantitative analysis of fusion index (I) of myogenic assay. (J-M) Western blotting (J) and quantitative analysis MyoD (K), MyoG (L), and MyHC (M) levels during myogenic induction. GAPDH was used as a loading control. (C and I) n = 3, three fields per sample were selected. Values are shown as mean ± SD. ***P < 0.001, student’s t-test. (E-F, K-M) n = 3. Values are shown as mean ± SD. *P < 0.05, **P < 0.01, ***P < 0.001, student’s t-test. (B) Scale bar = 50 μm. (H) Scale bar = 200 μm.

**
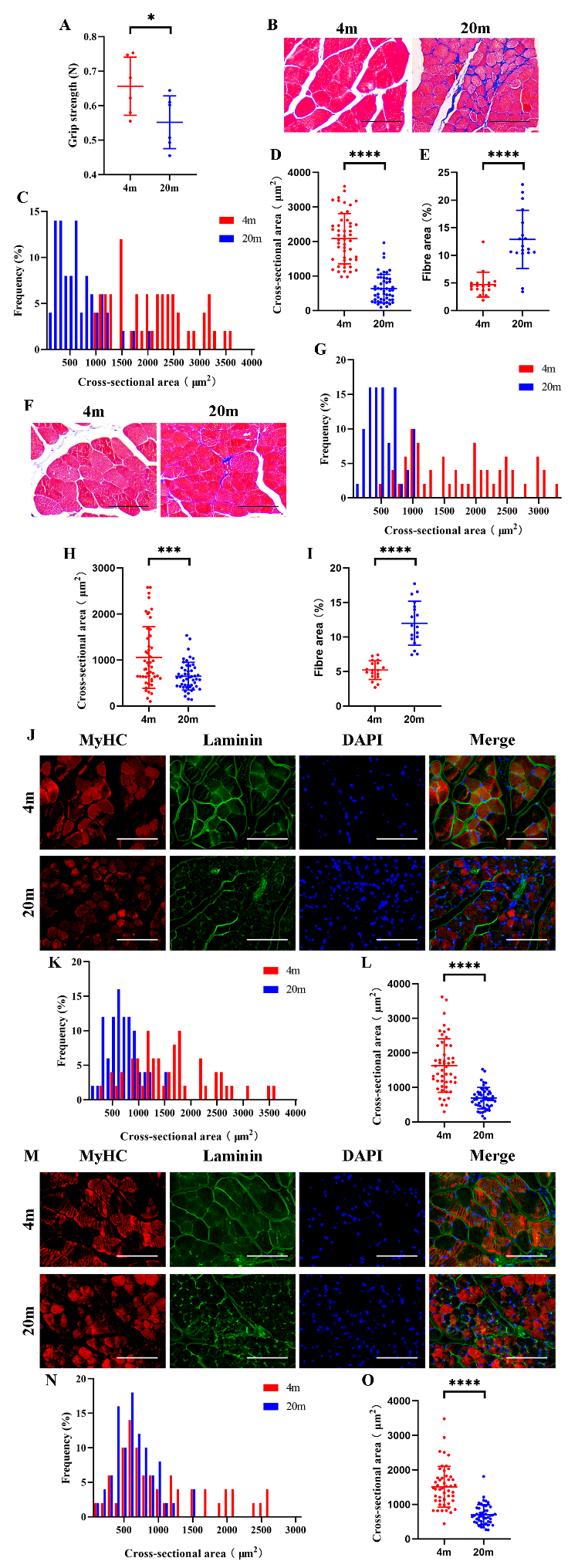
**

**Supplemental Fig. 3. Obvious muscle atrophy was detected in 20-months-old mice.** (A) 20-month-old mice display a lower hindlimb grip strength compared to 4-month-old mice. (B-E) Representative HE staining (B) and quantitative analysis of CSA (C and D) and fibre area (E) of MG. (F-I) Representative HE staining (F) and quantitative analysis of CSA (G and H) and fibre area (I) of TA. (J-L) Representative immunofluorescence staining of MyHC (J) and quantitative analysis of CSA of type II fibres (K and L) of MG. (M-O) Representative immunofluorescence staining of MyHC (M) and quantitative analysis of CSA of type II fibres (N and O) of TA. (A) n=6. Values are shown as mean ± SD. *P < 0.05, student’s t-test. (C, D, G, H, K, L, N, and O) n=6, 50 myotubes were assessed. Values are shown as mean ± SD. ***P < 0.001, ****P < 0.0001, student’s t-test. (E and I) n = 6, three fields per sample were selected. Values are shown as mean ± SD. ****P < 0.0001, student’s t-test. (B, F, J, and M) Scale bar = 50 μm.

**
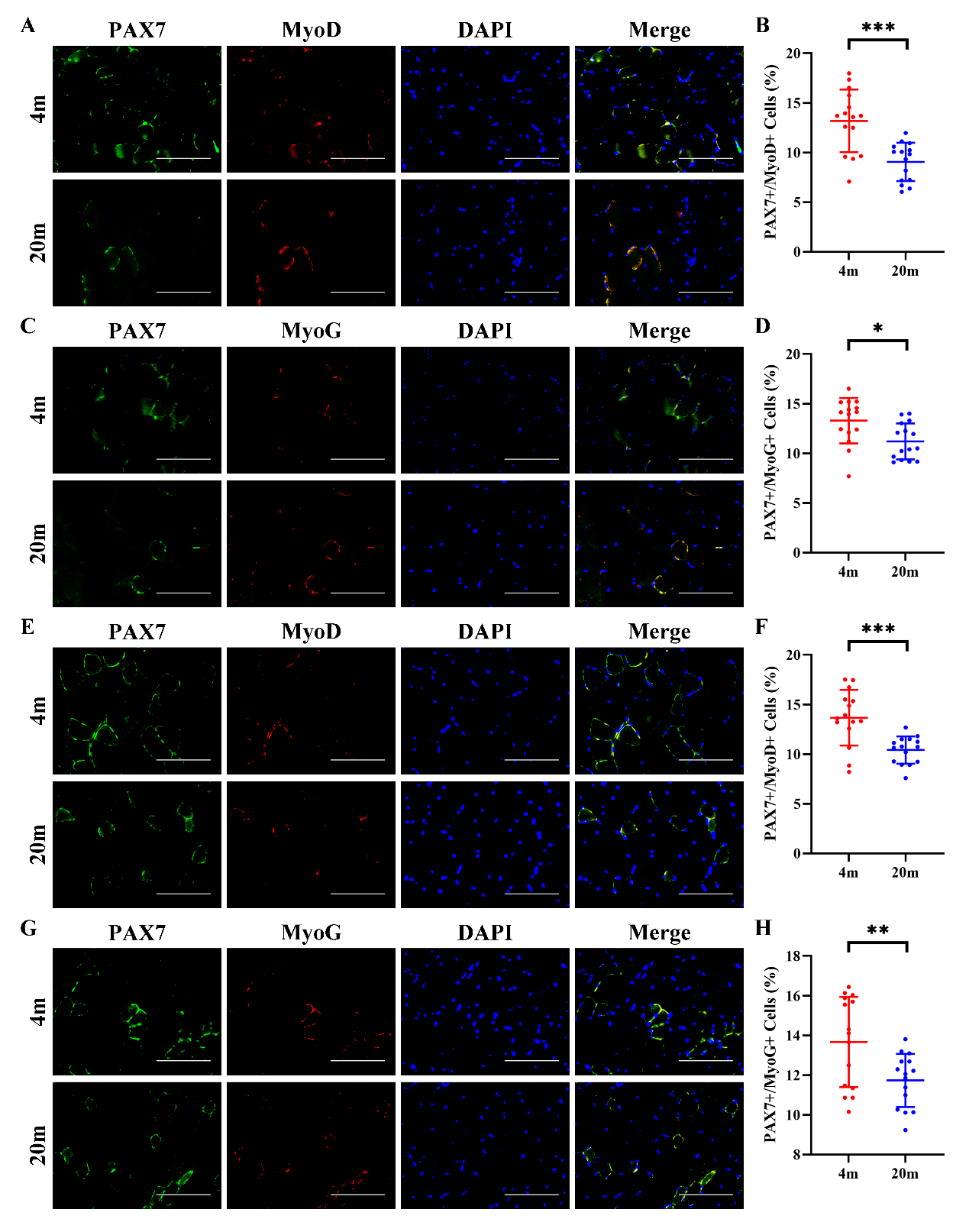
**

**Supplemental Fig. 4. SCs in 20-months-old mice showed significantly declined ability to myogenic differentiation.** (A and B) Representative immunofluorescence staining of PAX7 and MyoD (A) and PAX7+/MyoD+ cells counting of MG (B). (C and D) Representative immunofluorescence staining of PAX7 and MyoD (C) and PAX7+/MyoD+ cells counting of TA (D). (E and F) Representative immunofluorescence staining of PAX7 and MyoG (E) and PAX7+/MyoG+ cells counting of MG (F). (G and H) Representative immunofluorescence staining of PAX7 and MyoG (G) and PAX7+/MyoG+ cells counting of TA (H). (B, D, F, H) n = 6, three fields per sample were selected. Values are shown as mean ± SD. *P < 0.05, **P < 0.01, ***P < 0.001, student’s t-test. (A, C, E, and G) Scale bar = 50 μm.

**
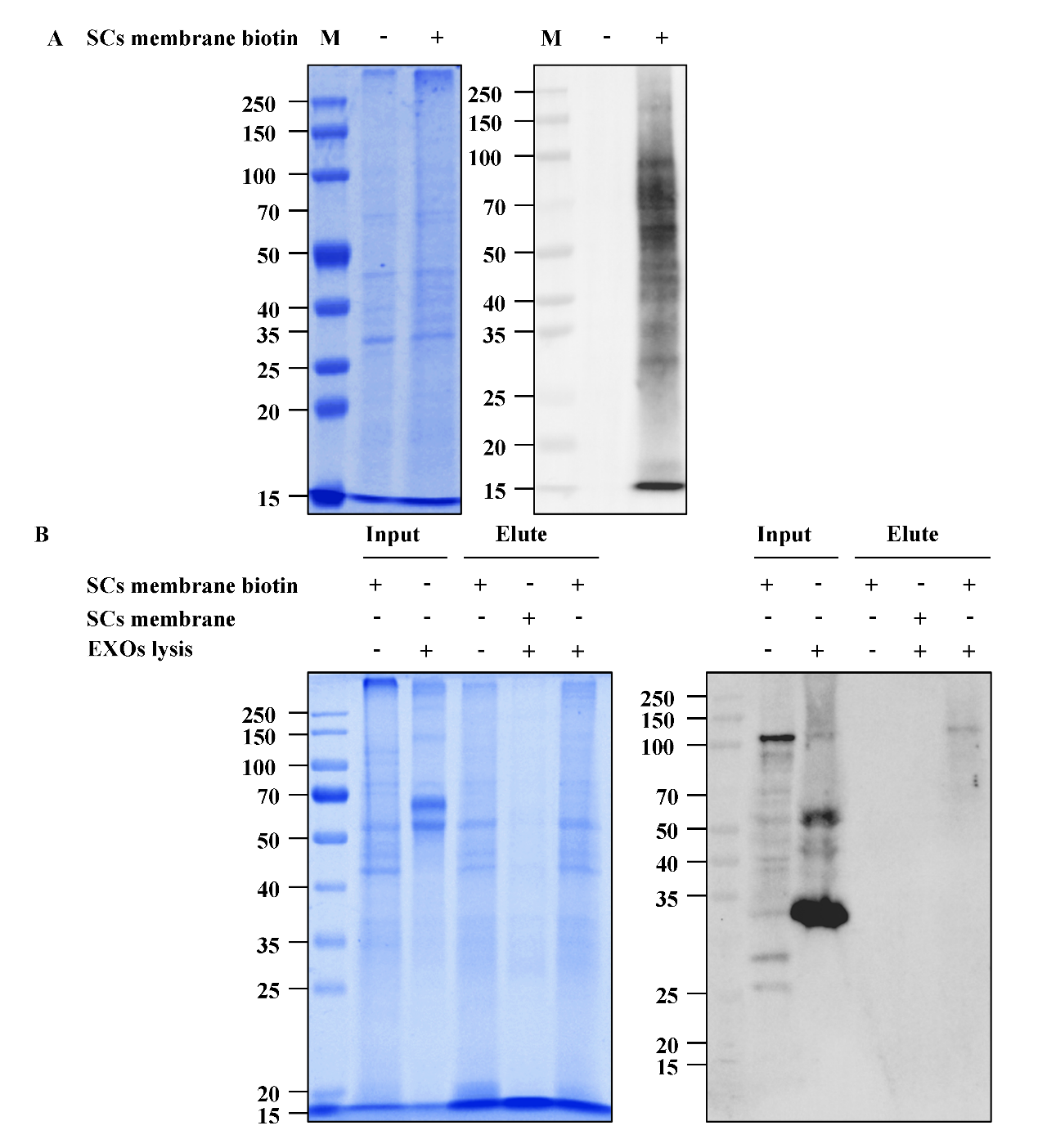
**

**Supplemental Fig. 5. CD81 on the membrane of BMSC-EVs could be bound to SCs membrane proteins, thus forming complex.** (A) SCs membrane proteins were labeled with biotin. (B) Biotin pulldown assay showed that CD81 on BMSC-EVs could be pulldowned by biotin labeled SCs membrane proteins.
